# Analysis of residential satisfaction in the conversion of Beijing’s stock buildings into rental housing

**DOI:** 10.1038/s41598-024-54081-1

**Published:** 2024-02-22

**Authors:** Xuefen Hu, Jian Suo, Ningbo Kou, Mengxue Wu, Shiyu Wang

**Affiliations:** The School of Architecture and Fine Art, Dalian University of Technology, Dalian, 116024 Liaoning People’s Republic of China

**Keywords:** Residential satisfaction, Influencing factors, Conversion rental housing, Young people, Functional conversion, Environmental sciences, Environmental social sciences, Psychology and behaviour, Sustainability

## Abstract

This paper investigates the residential satisfaction levels of tenants living in rental housing converted from non-residential stock buildings in Beijing. A stratified random sampling method was used to select 353 tenants from five apartments based on the plan form and location of the units for a structured questionnaire survey. The results of a hierarchical regression analysis indicated that subjective attributes were more influential in determining residential satisfaction than the objective physical and demographic attributes of the apartments. Within the five dimensions of subjective attributes, the "interior space" dimension had the greatest impact on predicting residential satisfaction. In addition, a one-way ANOVA analysis showed that the floor plan of the apartments also played a significant role in determining residential satisfaction, S-type and office park-type (Converted from an office park) layouts received the highest satisfaction ratings. This research provides valuable insights for revitalizing non-residential stock buildings and offers theoretical support for converting more non-residential stock buildings into rental housing in the future.

## Introduction

High property prices, coupled with an increasing number of floating populations and graduates, have exacerbated the problem of renting in large cities. According to the 7th National Population Census, the total number of China's floating population has increased significantly, with an average annual growth rate of 6.97%. Additionally, the number of college graduates is gradually increasing, with the nationwide number of college graduates already reaching 10.76 million in 2022, an increase of 1.67 million year-on-year, which is a record high in both scale and increment. The yearly increase in floating populations has become an inevitable trend in future social development, while high housing prices have deterred many of them. Increasing investment in rental housing can alleviate the housing difficulties faced by floating populations.

In the real estate sector, the vacancy rate of commercial premises in large urban centers continues to rise. According to broad-based inventory calculations, the depletion cycle for office and commercial premises exceeds the cap by 111 and 133% respectively in 2020, with a serious oversupply. In 2021, the General Office of the State Council issued the Opinions on Accelerating the Development of Secure Rental Housing, which explicitly allows the conversion of unused non-residential buildings into secure rental housing. Many cities have followed the national policy and converted part of the non-residential stock into rental housing. A number of projects have been implemented, which on the one hand provide an alternative way to solve the problem of non-residential building stock, and on the other hand enable unused resources to release government dividends through policy supply and alleviate the pressure of housing for the floating population. The implementation of the above policies has to a certain extent alleviated the problem of affordable housing for young people, but the living quality of the converted buildings has become a topic of concern.

In order to gain a better understanding of the current living conditions of the tenants, the study uses residential satisfaction as the main indicator to explore the main influencing factors that affect tenants' residential satisfaction. Satisfaction is the degree to which individuals' perception and evaluation of their residential environment, and it has been extensively studied and discussed in various disciplines such as psychology^1^, sociology^2^, and geography^3^. However, there is limited research on the satisfaction of rental housing with mobility characteristics in the domestic context. This paper primarily relies on the successful cases of converting non-residential buildings into rental housing, as announced by the Beijing Municipal Commission of Housing and Urban–Rural Development, to select five typical cases as research objects based on factors such as different administrative districts and architectural layout. This selection aims to enhance the representativeness and universality of the research results. Through the analysis and summary of these cases, this study aims to provide guidance and reference for the conversion of stock buildings into rental housing in other cities, ultimately improving residential satisfaction and the quality of rental housing. The basic information of the selected cases is presented in Table 1. Meanwhile, this study is based on Amerigo’s conceptual framework of cognitive, affective and behavioural aspects^4^, and takes into account the current situation of rental housing in China to provide a reference for the design of future conversions of non-residential stock into rental housing.

**Table 1:** Basic information on the five cases (*Source*: Author’s Own Compilation).

Case 1	Linear-type	
Location	Wenhuiyuan West Road, Haidian District, Beijing	
Original function	The building used to be an Express Hotel, a bathing centre and a KTV	
Specific introduction	The building is located in the north of Beijing’s second ring road, near the Xizhimen Metro Station, with convenient transportation. It is bordered on the north by commercial and office buildings, and is surrounded on the east and west by residential areas. The main structure of the building consists of an L-type ground floor and a straight standard floor. The L-type part retains its original function (commercial space on the ground floor), while the straight part has been renovated to create residential space	
Illustrations (standard floor plan, general plan, photographs)		
	
Case 2	U-type	
Location	Shunping Road, Shunyi District, Beijing	
Original function	The building was originally a commercial office building	
Specific introduction	The original building was U-type, in response to Beijing’s announced renovation policy, the central part was renovated into rental housing, while the two sides retained their original function (office space). The north and west sides of the building are commercial buildings, the south side is undeveloped land, and the east side is green space. As a commercial building renovation, the floor height is relatively high, and most of the rooms have been converted to loft style. This provides a good living environment for the nearby office population	
Illustrations (standard floor plan, general plan, photographs)		
	
Case 3	S-type	
Location	Tianzhu Town, Shunyi District, Beijing	
Original function	The building was originally a commercial and financial building	
Specific introduction	The building has an S-type layout, with all areas except for the public entertainment zone and auxiliary facilities converted into rental housing. There are over 600 units available, encompassing various types such as lofts, single-floor apartments, and one-bedroom units. The building is bordered by commercial buildings on the north and east sides, with green space on the west side and undeveloped land on the south side	
Illustrations (standard floor plan, general plan, photographs)		
	
Case 4	Clip-type	
Location	Anzhen Xili, Chaoyang District, Beijing	
Original function	The building was originally an office building and later converted into a hotel	
Specific introduction	This building is a typical old office building from the 1990s, with cramped and crowded rooms that lack natural light and ventilation, giving a sense of oppression and confinement. Thanks to the implementation of policies, the integration of new and old building spaces, the reconfiguration of functional areas, and the optimization of the building layout, the building has been transformed into rental housing, injecting new vitality into the process of urban renewal for old structures. The north side of the building is bordered by schools and a commercial area, while the south side is a residential area, the west side is a park, and the east side is commercial buildings	
Illustrations (standard floor plan, general plan, photographs)		
	
Case 5	office park-type	
Location	Guanzhuang Road, Chaoyang District, Beijing	
Original function	This building was originally an office and dormitory building in the compound of a state-owned enterprise	
Specific introduction	The original park was once a park of a state-owned enterprise and became an idle asset during the process of corporate transformation. The inner courtyard is enclosed by an office building on the north side, employee dormitories on the south side, and a cafeteria and bathroom building on the west side, forming a square central plaza. After the renovation, the north and south buildings have been transformed into rental apartments, while the cafeteria and bathrooms have been adjusted to serve as co-working spaces and a public kitchen. The park is conveniently located near a subway station, with easy transportation access. On the south, north, and east sides are commercial buildings, while the west side is a residential area, with the CBD located not far away	
Illustrations (standard floor plan, general plan, photographs)		
	

### Literature review

The study of residential satisfaction first emerged in the late 1950s, with researchers proposing the theory of family housing adaptation based on residents’ adaptation to their living environment in 1956^5^. Similarly, in 1975, Morris defined housing satisfaction as a dynamic process and introduced the theory of family housing adjustment^6^. Subsequently, others have explored the link between psychology and residential satisfaction, proposing the theory of psychological constructs^7^.

However, early literature did not explicitly propose a theoretical framework for residential satisfaction until a study in 1989 mentioned that residential satisfaction can be divided into three dimensions: cognitive, affective, and intentional. These dimensions consist of various factors that can interact with each other and collectively influence residents’ satisfaction with their living environment^8^. Amerigo’s research supported Francescato’s perspective and provided a summary definition of residential satisfaction: it is the result of residents’ evaluation of the objective attributes of their living environment, and he believes that satisfaction is the affective state that residents develop towards their living environment. Based on this viewpoint, a model of residential satisfaction was established^9^. Amerigo’s conceptual model proposes that subjective, objective, and demographic attributes have an impact on residential satisfaction, and he argues that residential satisfaction is a multi-dimensional construct.

In related studies, many scholars have focused on one part of the above dimensions. First, in terms of demographic attributes, age, gender, education, and income all influence residential satisfaction. For example, there is a positive correlation between age and residential satisfaction^10^; females are more likely to express dissatisfaction with housing compared to males^1^; and shorter commute times increase residential satisfaction^11^. Secondly, in terms of objective attributes, the architectural stylistic characteristics, the house size and the presence or absence of kitchens can also affect residential satisfaction, for example by exploring the impact of the building’s plan form on residential satisfaction^12^; the number of bedrooms and the size of the kitchen and dining room show a significant positive correlation with residential satisfaction^13^. Finally, there are additional dimensions in subjective attributes that affect residential satisfaction, such as interior space, common space, property management, social interaction, and neighborhood environment. For example, exploring the relationship between accessibility to public transportation and satisfaction^14^; the distance of schools, shopping centers and medical services from the location where one lives affects residential satisfactio^13^; it has also been suggested that satisfaction with the community environment and room interiors are important predictors of residential satisfaction^15^.

However, most studies on residential satisfaction have focused on Western countries. In contrast, domestic studies on the evaluation of residential satisfaction primarily focus on factors extracted from standards and norms, such as applicability, safety, and cost-effectiveness^16^. These studies lack the exploration of the correlation between individuals and their living environment. In the past decade, the research on residential satisfaction has mainly targeted residential areas, with a concentration on specific groups like married individuals and older adults. For example, there have been studies exploring the residential satisfaction of elderly individuals with different family compositions^17^. Some studies have investigated the residential satisfaction of residents in four representative neighborhoods in Shenzhen, considering various factors such as public transportation, educational resources, and green environments^18^. Additionally, there are studies that focus on individuals residing in traditional alleyways, impoverished spaces in new urban areas^19^, public rental housing^20^, and traditional villages^21^, discussing their residential satisfaction.

Since the introduction of the Opinions on Accelerating the Development of Secure Rental Housing policy in 2021 to address the housing problems of young people, research on rental housing^22^ and secure housing^23^ has begun to increase. In the context of this policy, this paper explores the factors influencing the residential satisfaction of converted rental housing by developing a hierarchical regression model to discuss the following issues:

1. What is the level of residential satisfaction of tenants in converted rental housing?
2. Is the attribute that best represents residential satisfaction a subjective attribute?
3. Do the objective attributes of apartments affect residential satisfaction?
4. Do all five subjective attribute dimensions examined in this study impact residential satisfaction?

## Research methodology

### Conceptual model of residential satisfaction

This study applied the conceptualization model proposed in Amerigo to study the interaction process between tenants and their living environment, which is considered as cognitive, affective and behavioral^4^. Based on this model, this study argues that residential satisfaction is influenced by demographic attributes, physical attributes of the apartment, and subjective attributes, and that individual attributes and physical attributes affect apartment satisfaction directly or indirectly through subjective attributes. The independent variables in this study are individual attributes, physical attributes, and subjective attributes, the dependent variable is residential satisfaction.

### Data source

**Figure 1:**
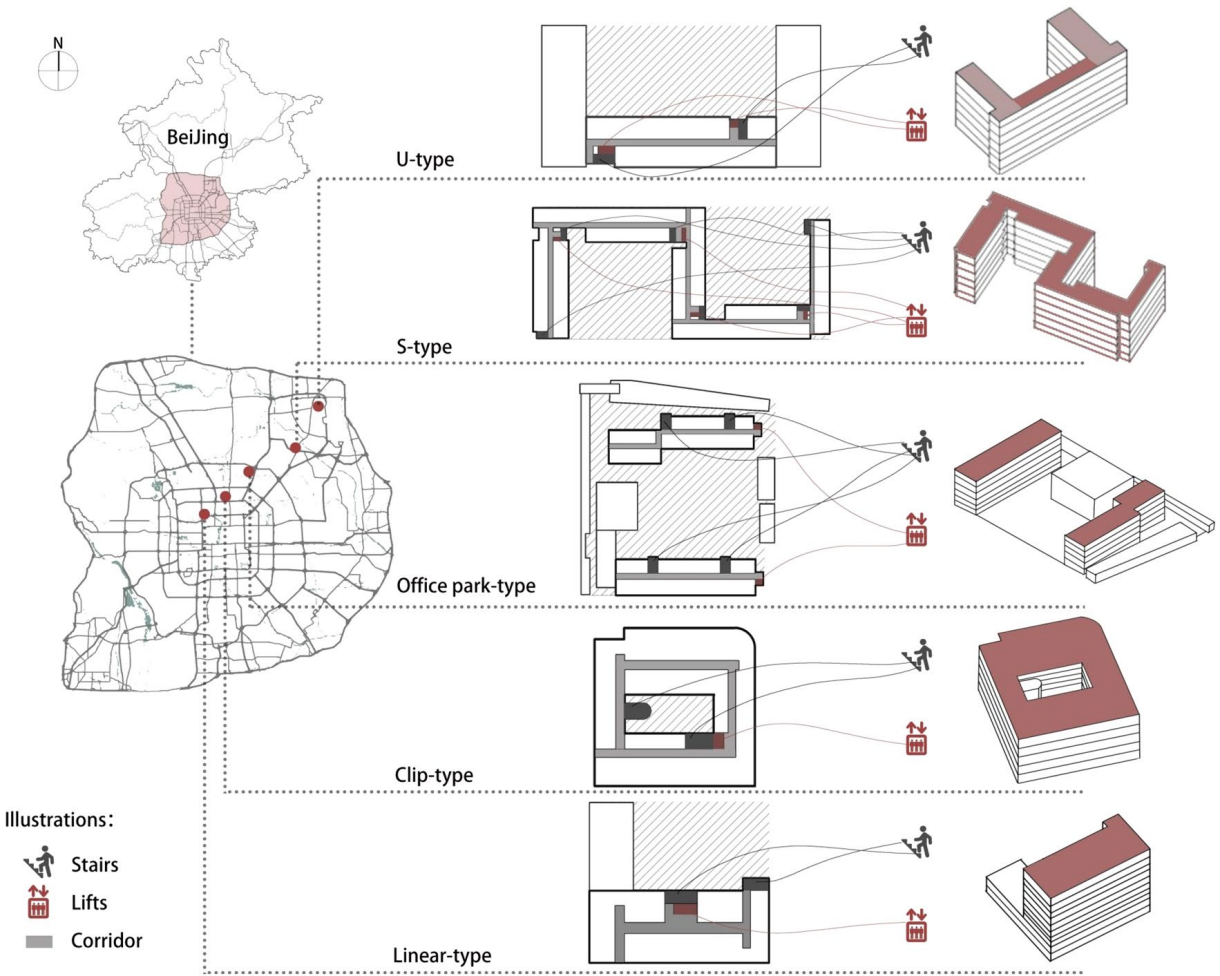
Map of apartment site selection. (Source: adapted from data visualisation platform aliyun.com (left), self-drawn (centre、right)).

This study investigates tenants of rental housing units converted from non-residential stock buildings in Beijing. Five converted rental housing types were selected based on the location and shape of the rental housing and other conditions to ensure the universality of the sample, and the specific locations and shapes are shown in Fig. 1. The respondents were selected from each of the rental flats with different shapes using a stratified random sampling method, and 362 questionnaires were finally returned, obtaining 353 valid questionnaires.

#### Sample size

The total sample size is derived from the sampling formula: n = P(1-P)/((e2/Z2) + (P(1-P)/N)) = 308.6≈309 (where P = 0.5; e = 0.05; Z = 1.96; N = 1570). he effective number of questionnaires meets the above requirements, and the specific sample size distribution is shown in Table 2.

**Table 2:** Basic Information of Surveyed Buildings and Sample Size Allocation. (*Source*: Author’s Own Compilation).

Number	Building form	Number of rooms	Number of rented rooms	Occupancy rate (%)	Sample size allocation	Actual number of collected questionnaires
1	Linear-type	263	258	98.1	60	69
2	U-type	306	301	98.4	61	65
3	S-type	501	402	80.2	80	89
4	Clip-type	324	310	95.7	52	61
5	Office Park-type	303	300	99	60	69

### Research variables

#### Independent variables

The independent variables consist of three parts, demographic variables, objective physical variables, and subjective variables. The attitude questions for the subjective variables are measured using a Likert scale with five ratings ranging from very dissatisfied (1) to very satisfied (5). For specific details, please refer to Table 3.

**Table 3:** Indicators of Independent Variables. (*Source*: Author’s Own Compilation).

Demographic variables	Gender (X1), age (X2), marital status (X3), education (X4), occupational category (X5), monthly rent as a proportion of monthly salary (X6), length of residence (X7), duration of participation in work (X8), commuting time (X9), and mode of commuting (X10)^24–29^
Objective physical variables	Plan form of the apartment (X11), room style (studio, loft, one-bedroom, two-bedroom) (X12), room size (X13), presence of a kitchen in the room (X14), presence of a gym, activity room, etc. in the flat (X15), presence of a hospitality area, gathering area, etc. in the flat (X16)^12,24,30,31^
subjective variables	Interior space, reasonableness of the functional layout of the room (X19A), width and depth (X19B), completeness of kitchen and bathroom facilities (X19C), interior decoration (X19D), storage space (X19E), use of electrical appliances (refrigerator, washing machine, air conditioning) (X19F), natural lighting, ventilation and illumination (X19G), comfort of temperature (X19H), noise control (X19I)^24,27,32–37^
	Common space, relationship between corridors and elevators (or stairs) (X20A), use of (communal) kitchens (X20B), use of public fitness and recreational areas (X20C), plan form of the apartment (X20D), location of entrance foyers (X20E)^12,14,24,32,38^
	Property management, maintenance and security services (X21A), cleaning services (X21B), Courier pick-up and storage (X21C), smart services (X21D)^24,28,30,32,34^
	Social interaction, interactions with other tenants in the apartment (X22A), sense of attachment and belonging to the apartment (X22B), events organized within the apartment complex (X22C), social security in the apartment and its surroundings (X22D)^24,30,31,33,39^
	Neighborhood environment, distance to medical facilities (X23A), distance to shopping malls, supermarkets, banks, etc. (X23B), distance to parks, squares, and other recreational areas (X23C), distance to bus stops or subway stations (X23D), convenience of parking facilities (X23E), accessibility design (X23F)^24,27,28,30–33,40^

#### Dependent variables

The dependent variable in this study is residential satisfaction, which includes the following three questions:

(Y18A) Are you satisfied with living here in general?
(Y18B) How likely is it that you will continue to live here?
(Y18C) How likely are you to recommend it to a friend?

The responses to the three questions above were measured using the Likert scale. For Question 1, a score of 1 represents "very dissatisfied" and a score of 5 represents “very satisfied.” For Question 2, a score of 1 represents “strongly inclined to move out immediately,” as individuals who express a desire to move out immediately are to some extent dissatisfied with their living environment, while a score of 5 represents "not inclined to move out." For Question 3, a score of 1 represents "strongly unwilling to recommend," and a score of 5 represents "strongly willing to recommend," indicating that individuals are satisfied with their living situation and would recommend it to others. The average score of each respondent on these questions is calculated as the dependent variable.

### Data analysis

The data were analyzed using SPSS 27.0 software. Firstly, descriptive statistics were used to analyse the profile of the respondents. Secondly, the five dimensions of the independent variables were reliability analysis, as well as the three questions that comprised the dependent variable. Thirdly, correlation analyses were conducted on all subjective variables with the dependent variable to ensure that the data could be continued for regression analysis. Fourthly, the data were processed using a hierarchical regression model to determine the effect of various factors on satisfaction. The first tier regression model included demographic and objective physical variables, resulting in model fit (R^2^) and predictors of satisfaction. The second level regression model included the variables used in the first regression analysis as well as subjective variables, which yielded the model fit (R^2^) and predictors of satisfaction. Finally, a one-way ANOVA was used to explore whether the form of the flat was an influential factor in residential satisfaction.

### Ethics review

This study has passed the review of the Biological and Medical Ethics Committee of Dalian University of Technology before starting, and the review was approved.

## Results

### Analysis of the basic attributes of the respondents (frequency analysis)

The profiles of the respondents are presented in Table 4. Firstly, the survey respondents were mostly young people aged 20–35, about a third was male while two-thirds were female, and a majority of them were unmarried. Secondly, most of the respondents’ monthly rent accounts for 20–40% of their monthly salary, and the commuting time was mostly less than 60 min, with public transportation such as buses and subways being the main mode of transportation. Finally, in terms of residential satisfaction, 62% of tenants were satisfied with their current living environment, 30% felt it was average, and fewer than 10% were dissatisfied with their living environment.

**Table 4:** Basic information on survey respondents. (*Source*: Author’s Own Compilation).

Characteristic	Categories	Frequency	% of Respondents
Gender	Male	123	34.844
	Female	230	65.156
Marital status	Unmarried	297	84.136
	Married	56	15.864
Age	Under 25 years old	131	37.11
	26–30	162	45.892
	31–35	51	14.448
	Over 36 years old	9	2.55
Proportion of monthly rent to monthly income	Under 20%	78	22.096
	20–40%	188	53.258
	40–60%	71	20.113
	Over 60%	16	4.533
Joined the workforce	Less than 1 year	46	13.031
	1-2years	102	28.895
	2-3years	76	21.53
	3-4years	58	16.431
	More than 4 years	71	20.113
Education level	Lower secondary and below	60	16.997
	High school	78	22.096
	University	186	52.691
	Postgraduate and above	29	8.215
One-way commute	Less than 30 min	169	47.875
	30–60min	160	45.326
	60–90min	22	6.232
	90 min or longer	2	0.567
Commute transportation mode	Bicycles/electric vehicles	94	26.629
	Bus/metro	195	55.241
	Private car	30	8.499
	Walking	34	9.632
Residential satisfaction	Very dissatisfied	3	0.85
	Unsatisfactory	27	7.649
	Neutral	104	29.462
	Satisfied	172	48.725
	Very satisfied	47	13.314
Total		353	100

### Reliability analysis

The SPSS reliability analysis is a data analysis method that measures the internal consistency of a questionnaire by calculating the correlation between the factors in the questionnaire. The overall reliability coefficient for the subjective variables X19A-X23F measured in this study was 0.936 and the reliability coefficients for each dimension are shown in Table 5.

**Table 5:** Reliability coefficients for each dimension. (*Source*: Author’s Own Compilation).

Variable name	Sample number	Number of factors	Cronbach’s α
Interior space	353	9	0.833
Common space	353	5	0.743
Property management	353	4	0.731
Social interaction	353	4	0.700
Neighborhood environment	353	6	0.760

In addition, we also conducted reliability analysis on the data of the three dependent variables that constitute residence satisfaction. The Cronbach’s α coefficient was 0.820, indicating high reliability, indicating that the dependent variables were intrinsically correlated, which verified Amerigo's claim that residence satisfaction is a dynamic process with cognitive, emotional and behavioral aspects. They are closely related^4^.

### Correlation analysis

The Pearson method was used to conduct correlation analysis on the subjective variables X19A–X23F with the dependent variable, and Table 6 shows that the correlation coefficient between most of the variables were greater than 0.4, indicating that they are moderately correlated, while electrical equipment such as refrigerators, washing machines, air conditioning use (X19F), noise control (X19I), distance from parkland and recreational areas (X23C) and paving and ramp design around the flats (X23F) showed weak correlations with the dependent variables.

**Table 6:** Correlation analysis of the subjective variables X19A-X23F with the dependent variable. (*Source*: Author’s Own Compilation).

Subjective variables	Correlation	Subjective variables	Correlation	Subjective variables	Correlation
X19A	0.720**	X20B	0.568**	X22C	0.436**
X19B	0.699**	X20C	0.406**	X22D	0.445**
X19C	0.574**	X20D	0.465**	X23A	0.495**
X19D	0.555**	X20E	0.527**	X23B	0.549**
X19E	0.548**	X21A	0.598**	X23C	0.353**
X19F	0.347**	X21B	0.480**	X23D	0.540**
X19G	0.527**	X21C	0.478**	X23E	0.482**
X19H	0.450**	X21D	0.423**	X23F	0.329**
X19I	0.324**	X22A	0.560**		
X20A	0.574**	X22B	0.520**		

** Significant correlation at the 0.01 level (two-tailed); * Significant correlation at the 0.05 level (two-tailed).

### Stratified regression

To explore the factors influencing residential satisfaction in rental housing, a hierarchical regression model was developed. In the first level of the regression model (control level), the influence of demographic and physical characteristics on Residential satisfaction was verified, with objective physical variables (X1-X10) and demographic variables (X11-X15) as control variables and the means of Y18A, Y18B and Y18C as dependent variables, as shown in Table 7. In the second level of the regression model (level 1), the independent variables include those from level 1 and all subjective variables (X19A-X23F), as shown in Table 8. The regression model underwent tests for multicollinearity and independent residuals, and all variables had variance inflation factors (VIF) less than 10, indicating no multicollinearity among the independent variables and meeting the statistical requirements.

**Table 7:** Regression model 1 of residential satisfaction (control level). (*Source*: Author’s Own Compilation).

Model1	Unstandardized coefficient		Standardized coefficient	t	P	95.0% confidence interval for B		VIF
	B	Std error	Beta			Lower-bound	Upper-bound	
Demographic variables								
X1	− 0.214	0.086	− 0.122	− 2.482	0.014**	− 0.383	− 0.044	1.033
X2	− 0.084	0.088	− 0.077	− 0.946	0.345	− 0.258	0.09	2.814
X3	− 0.527	0.16	− 0.231	− 3.295	0.001***	− 0.841	− 0.212	2.092
X4	0.472	0.083	0.465	5.682	0.000***	0.309	0.635	2.851
X5	− 0.05	0.063	− 0.044	− 0.792	0.429	− 0.175	0.074	1.303
X6	− 0.07	0.067	− 0.065	− 1.04	0.299	− 0.202	0.062	1.652
X7	0.15	0.083	0.101	1.819	0.070*	− 0.012	0.313	1.315
X8	0.093	0.047	0.148	1.965	0.050*	0	0.185	2.419
X9	− 0.201	0.068	− 0.153	− 2.952	0.003***	− 0.335	− 0.067	1.14
X10	0.022	0.049	0.023	0.458	0.648	− 0.073	0.118	1.064
Objective physical variables								
X11	0.029	0.033	0.048	0.88	0.379	− 0.036	0.094	1.284
X12	− 0.003	0.079	− 0.002	− 0.035	0.972	− 0.159	0.153	1.706
X13	0.032	0.1	0.017	0.316	0.752	− 0.166	0.229	1.263
X14	0.263	0.174	0.132	1.518	0.13	− 0.078	0.605	3.238
X15	0.021	0.13	0.013	0.164	0.87	− 0.234	0.276	2.495
R^2^	0.209							
Adjusted R^2^	0.174							
F value	F(15, 353) = 5.95, P = 0.000***							
△R^2^	0.209							
△F value	F(15, 353) = 5.95, P = 0.000***							

Dependent variable mean residential satisfaction.

*, **, *** indicate significant at P < 0.1, P < 0.05, P < 0.01 levels respectively.

**Table 8:** Regression model 2 of residential satisfaction (level 1). (*Source*: Author’s Own Compilation).

Model2	Unstandardized coefficient		Standardized coefficients	t	P	95.0% confidence interval for B		VIF
	B	Std error	Beta			Lower-bound	Upper-bound	
Demographic variables								
X1	0.025	0.051	0.014	0.497	0.62	− 0.075	0.126	1.19
X2	− 0.079	0.051	− 0.073	− 1.556	0.121	− 0.179	0.021	3.059
X3	− 0.017	0.095	− 0.007	− 0.178	0.859	− 0.203	0.169	2.406
X4	− 0.073	0.054	− 0.072	− 1.338	0.182	− 0.18	0.034	4.02
X5	− 0.02	0.037	− 0.017	− 0.533	0.594	− 0.092	0.053	1.444
X6	0.026	0.039	0.024	0.681	0.496	− 0.05	0.102	1.802
X7	0.079	0.048	0.053	1.645	0.101	− 0.016	0.174	1.47
X8	0.032	0.027	0.051	1.18	0.239	− 0.021	0.086	2.663
X9	− 0.031	0.04	− 0.023	− 0.772	0.44	− 0.109	0.047	1.268
X10	0.011	0.028	0.011	0.396	0.692	− 0.043	0.065	1.129
Objective physical variables								
X11	− 0.07	0.024	− 0.116	− 2.951	0.003***	− 0.116	− 0.023	2.165
X12	0.015	0.048	0.012	0.308	0.758	− 0.08	0.109	2.056
X13	0.121	0.058	0.066	2.097	0.037**	0.007	0.234	1.372
X14	0.005	0.126	0.003	0.043	0.966	− 0.242	0.253	5.588
X15	− 0.11	0.099	− 0.065	− 1.117	0.265	− 0.305	0.084	4.768
Subjective variables								
Interior space								
X19A	0.202	0.046	0.209	4.434	0.000***	0.112	0.292	3.114
X19B	0.165	0.038	0.194	4.323	0.000***	0.09	0.24	2.809
X19C	0.129	0.033	0.139	3.881	0.000***	0.064	0.195	1.8
X19D	0.095	0.036	0.114	2.656	0.008***	0.025	0.165	2.58
X19E	0.029	0.032	0.036	0.894	0.372	− 0.034	0.092	2.244
X19F	-0.047	0.031	− 0.05	− 1.508	0.133	− 0.108	0.014	1.546
X19G	0.071	0.033	0.078	2.13	0.034**	0.005	0.137	1.894
X19H	0.029	0.038	0.029	0.758	0.449	− 0.046	0.103	2.016
X19I	− 0.006	0.032	− 0.007	− 0.197	0.844	− 0.07	0.057	1.602
Common space								
X20A	0.121	0.036	0.121	3.344	0.001***	0.05	0.193	1.835
X20B	0.02	0.038	0.02	0.527	0.598	− 0.055	0.095	2.091
X20C	0.007	0.029	0.009	0.24	0.811	− 0.05	0.064	1.78
X20D	0.054	0.03	0.062	1.816	0.070*	− 0.005	0.113	1.639
X20E	0.033	0.034	0.036	0.99	0.323	− 0.033	0.1	1.854
Property management								
X21A	− 0.003	0.037	− 0.003	− 0.08	0.936	− 0.076	0.07	2.416
X21B	0.07	0.028	0.083	2.458	0.015**	0.014	0.126	1.584
X21C	0.028	0.036	0.031	0.767	0.444	− 0.043	0.098	2.243
X21D	− 0.054	0.034	− 0.06	− 1.586	0.114	− 0.121	0.013	1.999
Social interaction								
X22A	− 0.012	0.043	− 0.013	− 0.278	0.781	− 0.096	0.072	2.929
X22B	0.079	0.033	0.088	2.42	0.016**	0.015	0.143	1.84
X22C	0.027	0.031	0.03	0.87	0.385	− 0.034	0.087	1.64
X22D	− 0.016	0.033	− 0.018	− 0.492	0.623	− 0.082	0.049	1.781
Neighborhood environment								
X23A	0.045	0.036	0.056	1.256	0.21	− 0.026	0.116	2.82
X23B	0.083	0.031	0.104	2.705	0.007***	0.023	0.143	2.071
X23C	-0.007	0.03	-0.009	− 0.25	0.802	− 0.066	0.051	1.951
X23D	0.094	0.034	0.103	2.762	0.006***	0.027	0.162	1.959
X23E	− 0.023	0.032	− 0.025	− 0.706	0.481	− 0.087	0.041	1.786
X23F	− 0.019	0.029	− 0.025	− 0.664	0.507	− 0.076	0.038	2.057
R^2^	0.78							
Adjusted R^2^	0.749							
D-W value	2.09							
F value	F(43, 352) = 25.421, P = 0.000***							
△R^2^	0.57							
△F value	F(28, 352) = 28.555, P = 0.000***							

Dependent variable mean residential satisfaction.

*, **, *** indicate significant at P < 0.1, P < 0.05, P < 0.01 levels respectively.

The first level of the regression model showed a p-value of 0.000*** for the objective physical and demographic variables strata, showing significance and rejecting the original hypothesis, therefore the model was valid, while the model performed poorly with a goodness of fit R^2^of 0.211, indicating that the objective physical and demographic variables had a small effect on the dependent variable. Despite the significance of the model, the predictive strength was low. Table 7 shows that the variables that appear significant at the P < 0.01 level are marital status (X3), educational attainment (X4) and commuting time (X9), the variable significant at the P < 0.05 level is gender (X1), and the variables significant at the P < 0.1 level are length of residence (X7) and duration of participation in work (X8).

The p-value in the second level regression model was 0.000***, showing significance and rejecting the original hypothesis, so the model was valid, while the model performed relatively well with a goodness of fit R^2^ of 0.78. This indicates that the subjective attributes generated by individuals towards rental apartments explain the satisfaction with living better than the objective physical attributes and demographic attributes of the apartments themselves. In the second level regression, the demographic attributes of the model are not significant, the objective physical attributes of apartment form (X11) and apartment size (X13) are significant, and most of the significance is reflected in the subjective attributes, Table 8 shows that the reasonableness of the functional layout of the room (X19A), the span and depth (X19B), the completeness of kitchen and bathroom facilities (X19C), the interior decoration (X19D), the natural lighting and ventilation (X19G), the location of corridors in relation to lifts (stairs) (X20A),the plan form of the apartment (X20D), the cleaning services (X21B), the sense of attachment and belonging to the apartment (X22B), the distance from shopping malls, supermarkets, banks, etc. (X23B), the distance from bus or underground stations (X23D) all had a significant effect.

### One-way ANOVA

In order to verify that different plan forms of the apartment have an effect on residential satisfaction, a one-way analysis of variance (ANOVA) was conducted with plan form as the independent variable and the mean of residential satisfaction as the dependent variable, as shown in Table 9. The ANOVA analysis in Table 10 showed a significance level of less than 0.05, indicating a statistically significant effect of apartment form on satisfaction. The homogeneity of variance test in Table 11 showed significance levels greater than 0.05, indicating that the requirement for homogeneity of variance was met. Based on the above results, it is meaningful to discuss the impact of different plan forms of the apartment on tenants' residential satisfaction in the following section.

**Table 9:** Descriptive statistic. (*Source*: Author’s Own Compilation).

	N	Mean	S.D	Std Error	95% confidence interval for mean		Minimum value	Maximum value
					lower-bound	upper-bound		
Linear type	69	3.74	0.816	0.098	3.54	3.94	1	5
U-type	65	3.12	0.82	0.102	2.92	3.33	1	5
S-type	89	3.96	0.811	0.086	3.78	4.13	2	5
Clip-type	61	3.61	0.781	0.1	3.41	3.81	1	5
Office park-type	69	3.75	0.715	0.086	3.58	3.93	2	5
Total	353	3.66	0.835	0.044	3.57	3.75	1	5

Dependent variable: mean residential satisfaction.

**Table 10:** ANOVA. (*Source*: Author’s Own Compilation).

	Sum of squares	df	Mean square	F	Sig
Between-groups	27.698	4	6.924	11.079	< 0.01
Within-groups	217.509	348	0.625		
Total	245.207	352			

Dependent variable: mean residential satisfaction.

**Table 11:** Homogeneity of Variance Test. (*Source*: Author’s Own Compilation).

	Levene’s test	Degree of freedom 1	Degree of freedom 2	Sig
Based on mean	0.498	4	348	0.737
Based on median	0.293	4	348	0.883
Based on median with adjusted degrees of freedom	0.293	4	344.704	0.883
Based on trimmed means	0.59	4	348	0.67

Dependent variable: mean residential satisfaction.

## Discussion

### General residential satisfaction

According to Table 4, it can be observed that approximately 62% of the tenants expressed satisfaction or high satisfaction with the living environment, while around 38% of the tenants reported an average or dissatisfied living experience. Demographic attributes, objective physical attributes, and subjective attributes all influence tenants’ residential satisfaction. The R2 value for the demographic and objective attribute levels is 20.9%, while the R2 value for the subjective attribute level is 78%, indicating that subjective attributes are better predictors of residential satisfaction. This finding is consistent with previous research findings^12^. Furthermore, among the five dimensions of subjective attributes, interior space exhibits the strongest significance, which is similar to the research conclusion of Mohit et al^13,15^.

### Demographic variables

In the first level of regression analysis, the factors that produce an effect are gender (X1), marital status (X3), educational qualification (X4), and length of commute (X9), the possible reasons for this are as follows: the gender factor (X1) has an impact on tenants’ residential satisfaction, with men being more easily satisfied with their living environment than women. This is because men prioritize the practicality and functionality of space, while women may have further needs for their living environment, such as comfort and attachment, resulting in higher satisfaction levels for men than women in the same environment. This is consistent with Rioux’s conclusion^1^. The marital status of tenants (X3) also has an impact on residential satisfaction, with unmarried individuals being more easily satisfied, while married couples’ residential satisfaction is lower than that of unmarried individuals. This is because the respondents mostly lived in single-room rental housing of 30–40 square meters, which often has problems such as limited space and poor privacy. Additionally, tenants' educational level (X4) also has an impact on residential satisfaction, with postgraduate tenants being more satisfied with their living environment compared to those with high school or undergraduate degrees. This is not surprising, as people with higher educational levels usually have higher social and economic status and higher income levels, making them more likely to obtain better housing conditions and thus improving their residential satisfaction. this conclusion is similar to Rioux’s research^1,10^. In addition to gender, marital status, and education, the length of commuting time (X9) also affects residential satisfaction, with longer commuting times leading to lower satisfaction. Most of the tenants in this study commuted within 60 min, indicating that their workplaces were relatively close to their homes, which suggests that the location of the converted rental housing surveyed in this study was reasonable. This conclusion supports previous research: the more convenient the transportation, the higher the satisfaction^11^. In contrast, age (X2), occupational category (X5), monthly rent as a percentage of monthly salary (X6), length of residence (X7), duration of participation in work (X8), and mode of commuting (X10) did not have an impact on residential satisfaction. The age factor (X2) is due to the fact that most of the people living here are of the same age group and there is little difference in their living environment and expectations. Occupation factor (X5) and salary (X6) are because most people believe that their income is sufficient to pay the rent. The length of residence (X7) and duration of participation in work (X8) factors are due to the fact that most of the occupants have been in residence for around 1–2 years. Lastly, the commuting mode factor (X10) is because the building was sited with accessibility to the surrounding area in mind.

In the first level of regression analysis, although a variety of demographic attributes had an effect on residential satisfaction, the R^2^ was only 20.9%, which is not an adequate explanation for the dependent variable. In contrast, the second level of regression analysis resulted in an R^2^ of 78%, at which point none of the demographic attributes had an effect on residential satisfaction. This shows that the effect of demographic attributes on residential satisfaction is small, which validates the point made in Amole’s article^12^.

### Objective physical variables

**Figure 2:**
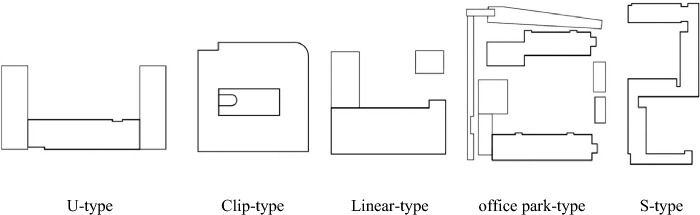
The five building forms explored in this study. (Source: Author's own drawing).

In terms of objective physical attributes, the plan form of the apartment (X11) has a significant impact on residential satisfaction, and different plan forms lead to differences in satisfaction. The five building forms explored in this study are shown in Fig. 2.The average satisfaction rankings of the five discussed building plan forms, from highest to lowest, are: S-type > office park-type > linear-type > clip-type > U-type (refer to Table 9). Possible reasons for the above sequencing are: the S-type plan has good lighting and views, allowing most rooms to have sufficient sunlight. Both semi-enclosed inner courtyards have more sunlit areas and better landscape configurations, creating a good outdoor space for residents to live in, with an overall higher level of living comfort. The office park-type plan is similar, but the north–south layout is designed so that half of the rooms lack sunshine, making the living satisfaction second to the S-type; in the linear-type plan, although there is better sunshine, three sides are facing the street, and the backyard is in the shadow, so the overall environmental space is obviously not as good as that of the former; in the clip-type plan, the internal patio is narrow, and there is interference in the internal view, lack of privacy, and noisy external environment. In the U-type plan, as there are commercial offices on both sides, there is serious visual interference, people are mixed, and security is poor, resulting in the lowest level of residential satisfaction. This finding is similar to Amole’s research results^12^.The study results indicate that room style (X12) had no significant impact, despite the inclusion of standard single rooms, lofts, and one-bedroom apartments in the survey. Tenants may place more emphasis on factors such as the apartment's location, size, and amenities, rather than just the room style. Therefore, the room style factor is relatively insignificant compared to other more important factors. In addition, the size of the living space (X13) had a significant impact on residential satisfaction, with larger spaces leading to higher tenant satisfaction. This finding is consistent with Ariffin’s research conclusions^41^. In fact, this is not surprising, as a larger living space can provide more storage, work, and relaxation space, making the residents' lives more comfortable and convenient.

### Subjective variables

The research results show that in the dimension of interior space, factors such as functional layout (X19A), room width and depth (X19B), completeness of kitchen and bathroom facilities (X19C), interior decoration (X19D), and natural lighting, ventilation, and lighting (X19G) all have an impact on residential satisfaction. Among the five dimensions, this dimension has the highest percentage of factors that affect residential satisfaction. As the interior space is the most private and direct living space for tenants, these factors are directly related to their quality of life and comfort. In addition, tenants can change the room layout or decoration style according to their preferences, thus better meeting their residential needs and improving overall satisfaction. Storage space (X19E) was found to have no significant impact, as tenants may adopt more storage strategies to maximize the use of limited storage space, and have made corresponding preparations and arrangements. Compared to storage space, tenants may be more concerned with factors such as area and layout. The noise factor (X19I) also did not emerge as a significant predictor, as property management imposes strict regulations on noise control, reducing the noise level in the apartments. Additionally, the use of electrical appliances (refrigerator, washing machine, air conditioning) (X19F) and the comfort of temperature (X19H) were found to have no impact, possibly because the rental apartments are well-equipped with electrical appliances.

In the dimension of common spaces, the factor of the relationship between corridors and elevators (or stairs) (X20A) influences residential satisfaction. If the relationship between corridors and elevators (or stairs) is reasonable, tenants can reach their destination more quickly and conveniently, improving travel efficiency and convenience, thus enhancing residential satisfaction. this has been confirmed by Baum's research^38^. The plan form of the apartment (X20D) also affects residents' residential satisfaction, possibly because residents prefer living in partially enclosed spaces, as supported by discussions on objective physical factors. The factor of public kitchen facilities (X20B) has not shown an impact, as most tenants are unmarried and rely on takeout for their meals, resulting in a lower demand for kitchen facilities. The factor of use of public fitness and recreational areas (X20C) has also not shown an impact, possibly due to the majority of tenants being commuters with limited leisure time, leading to minimal utilization of these facilities, as indicated by the survey.

In the dimension of property management, only the cleanliness service factor (X21B) has an impact on residential satisfaction. This is because sufficient cleanliness services can ensure the cleanliness and hygiene of the housing, improve the quality of the living environment, and enhance the quality of life of residents. this finding is consistent with Hui's study^39^. However, factors such as maintenance and security services (X21A), courier pick-up and storage (X21C), and smart services (X21D) did not show significant effects. The possible reason for this is that safety hazards or maintenance issues in newly converted housing have not yet emerged. This finding contrasts with Mohit's study, which highlighted the significant impact of property management (maintenance, security, etc.) on individuals' quality of life and residential satisfaction^33,42^.

In the dimension of social interaction, the sense of belonging and attachment to the residence (X22B) influences individuals’ residential satisfaction. If tenants can establish stable living habits based on their environment, they will develop emotional identification and attachment to the environment. This can improve their quality of life and convenience, leading to higher residential satisfaction. this finding is consistent with the research by Lee^43^. Another study also mentions a positive correlation between personal place attachment and residential satisfaction, highlighting that increasing place acceptance can enhance attachment^44^. Factors such as neighborly interaction (X22A) and collective activities (X22C) have not shown any significant impact. The reason could be the increased social distancing among tenants due to the influence of the COVID-19 pandemic in recent years, which has greatly reduced the opportunities and frequency of neighborly interaction and collective activities. The factor of social security in the apartment and its surroundings (X22D) has also not demonstrated any influence, possibly due to the crucial role played by security services in the apartment, prohibiting unauthorized access and improving the safety of the living environment.

In the dimension of neighborhood environment, factors that affect residential satisfaction include the proximity to shopping malls, supermarkets, banks, and other amenities (X23B), as well as the distance to bus stops or subway stations (X23D). This is because the distance to basic living facilities can affect tenants' transportation costs and time costs. If these facilities are located near the residential area, they can save on transportation costs and time, thereby improving the quality of life and residential satisfaction. The results showed that the distance to medical facilities (X23A) did not have an impact, as the tenants were primarily young and healthy with infrequent visits to medical buildings. The distance to parks, squares, and other recreational areas (X23C) also did not have an influence, possibly because younger individuals are less likely to engage in leisure activities such as strolling in parks compared to older adults. Furthermore, the convenience of parking facilities (X23E) and accessibility design (X23F) had no impact, likely due to the fact that the tenants were mainly young renters without private vehicles, making parking facilities and accessibility factors irrelevant as predictive variables.

## Conclusion

Under the guidance of existing national policies, this study investigated the residential satisfaction of tenants living in rental housing converted from non-residential stock buildings in Beijing. Relevant factors affecting residential satisfaction were analyzed and the research questions were addressed. Firstly, 62% of tenants were satisfied with their current living environment, 30% felt average, and less than 10% were dissatisfied with their living environment. Overall, the model of converting non-residential stock into rental housing is feasible. Secondly, subjective attributes were more likely to explain residential satisfaction than objective physical attributes and demographic attributes, which validates Amole’s findings^12^. Thirdly, among the objective physical attributes, the plan form and room size of the apartments had a significant impact on residential satisfaction, with tenants preferring layouts that offer larger interior spaces and better privacy. Fourthly, within the five subjective dimensions studied, factors related to the interior space dimension had the highest proportion of influence on satisfaction. Additionally, certain factors within the other four dimensions, such as cleaning services, sense of belonging, distance to shopping centers, and distance to public transportation stations (subway stations), also impacted residential satisfaction.

The aforementioned results are influenced by multiple factors. Firstly, the surrounding amenities and convenient transportation contribute to most tenants being able to address the issue of proximity to work. However, due to the original non-residential nature of the buildings, there are certain limitations in functional conversion, such as room orientation and depth. Additionally, the surrounding environment of the buildings also exhibits certain deficiencies, such as noise, lack of privacy, and mixed pedestrian traffic. Therefore, instances of dissatisfaction are also present. Secondly, respecting the subjective demands of residents and maximizing the fulfillment of their practical living needs can compensate for the deficiencies in objective conditions and achieve relative residential fairness. The demands of tenants can be expressed through the satisfaction with various subjective attributes, thus indicating that subjective attributes better represent the level of residential satisfaction. Thirdly, there has been a significant improvement in the living standards of young people, as they are pursuing a more comfortable and personalized lifestyle, with an increased emphasis on the comfort and privacy of their living spaces. Fourthly, the interior space serves as the most crucial area for tenants to relax, rest, and engage in recreational activities outside of work, meeting both material and spiritual needs. Additionally, tenants have higher expectations for the livability of the environment, such as convenient transportation and well-equipped facilities. Therefore, in the process of converting stock buildings into residential buildings, a reasonable site selection is of paramount importance. It is crucial to fulfill the commuting requirements of the majority of tenants as much as possible, which can effectively reduce the cost of living and enhance the quality of life. Moreover, conducting surveys to understand the living needs of residents and actively engaging in communication with them are indispensable. Lastly, further optimizing the design of the interior space to create diverse living spaces that cater to the personalized needs of tenants is essential. Furthermore, emphasizing the improvement of services and enhancing the livable conditions of the surrounding environment should also be considered.

According to statistics from Davis Company on office vacancy rates, in China's first-tier cities, the vacancy rate in Shenzhen is 24.4%, in Shanghai it is 15.9%, and in Beijing it is 16.8%, all of which significantly exceed the international warning line of 10%. In first-tier cities, it is difficult to develop new plots in the city center to construct rental housing and solve the housing problem for young people. Converting stock buildings to residential use is an inevitable trend for future urban development. This study has certain limitations as it only focuses on conversion cases in Beijing and does not make a comparative study of practices in other cities. However, the study provides valuable theoretical support and design references for converting non-residential stock buildings into rental housing in other cities.

## Data Availability

The data source of this article is from on-site research. If you need it, I am happy to provide the research data we have collected. The datasets used and analysed during the current study available from the corresponding author on reasonable request.
